# A contemporary case series of Fournier’s gangrene at a Swiss tertiary care center—can scoring systems accurately predict mortality and morbidity?

**DOI:** 10.1186/s13017-018-0187-0

**Published:** 2018-06-22

**Authors:** C. Wetterauer, J. Ebbing, A. Halla, R. Kuehl, S. Erb, A. Egli, D. J. Schaefer, H. H. Seifert

**Affiliations:** 1 Department of Urology, University Hospital Basel, Spitalstr. 21, 4031 Basel, Switzerland; 2 Division of Infectious Diseases and Hospital Epidemiology, University Hospital Basel, University Basel, Basel, Switzerland; 3 Division of Clinical Microbiology, University Hospital Basel, University Basel, Basel, Switzerland; 4 Applied Microbiology Research, Department of Biomedicine, University Basel, Basel, Switzerland; 5 Department of Plastic, Reconstructive, Aesthetic and Hand Surgery, University Hospital Basel, University Basel, Basel, Switzerland

**Keywords:** Fournier’s gangrene, Morbidity, Mortality, FGSI, LRINEC, NLR

## Abstract

**Background:**

Fournier’s gangrene (FG) is a life-threatening infection of the genital, perineal, and perianal regions with a morbidity range between 3 and 67%. Our aim is to report our experience in treatment of FG and to assess whether three different scoring systems can accurately predict mortality and morbidity in FG patients.

**Methods:**

All patients that were treated for FG at the Department of Urology of the University Hospital Basel between June 2012 and March 2017 were included and assessed retrospectively by chart review. Furthermore, we calculated Fournier’s Gangrene Severity Index (FGSI), the Laboratory Risk Indicator for Necrotizing Fasciitis (LRINEC), and the neutrophil–lymphocyte ratio (NLR) in every patient and assessed whether those scores correlate with the patients’ morbidity and mortality.

**Results:**

Twenty patients were included, with a median (IQR) age of 66 (46–73) years. Fifteen of twenty (75%) patients required treatment on an intensive care unit, and three died (mortality rate: 15%). The mean FGSI, LRINEC, and NLR scores were 13.0, 9.3, and 45.3 for non-survivors and 7.7, 6.5, and 26 for survivors, respectively. None of the risk scores correlated significantly with mortality; however, all three significantly correlated with infection- and surgically-induced morbidity.

**Conclusions:**

In our series, Fournier’s gangrene was associated with a mortality rate of 15% despite maximum multidisciplinary therapy at a specialized center. All risk scores were able to predict the morbidity of the disease in terms of local extent and the required surgical measures.

## Background

**Fig. 1:**
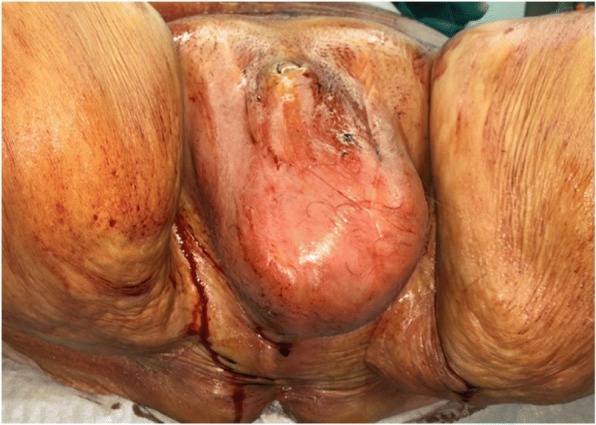
Classic clinical presentation of Fournier’s gangrene with scrotal/perineal swelling, tenderness on palpation, and poorly demarcated erythema, yet no visible necrosis of the skin

Fournier’s gangrene (FG) is a life-threatening infection of the genital, perineal, and perianal regions first described by Fournier in 1883 [1]. The male to female ratio is reported as 10:1 [2], with an incidence of 1:7500 to 1:750,000 [3]. Conditions leading to decreased host immunity and thus rapid spread of infection are considered predisposing factors [3]. FG is a polymicrobial aerobic and anaerobic infection caused by three or more pathogens and therefore classified as type 1 necrotizing tissue infection [4]. *Bacteroides fragilis* is the most commonly isolated anaerobic bacterial pathogen [5, 6], while *Escherichia coli* and *Enterococcus faecalis* represent the most commonly isolated aerobic pathogens [7]. In patients with diabetes mellitus, *Streptococcus* spp., *Staphylococcus* spp., and aerobic flora are regularly detected [8]. The combination of aerobic and anaerobic bacteria can lead to the production of several enzymes, such as collagenases and heparinases, which may result in tissue destruction and rapid progression of the infection. Thus, the combination of less pathogenic bacteria can develop high virulence in immunocompromised patients [2]. The FG diagnosis is based on clinical and laboratory findings. Patients usually present with classical signs of soft tissue infections of the scrotum, perineum, and/or perianal areas (Fig. 1). Additionally, gangrenous or necrotic tissue and skin areas may be visible. Leukocyte and C-reactive protein levels are usually elevated. Imaging can be helpful if the diagnosis is uncertain. Early diagnosis is essential for the outcome, yet diagnosis is often delayed in this relatively rare disease, which in turn results in a delayed treatment start [9]. Optimal multimodal treatment consists of hemodynamic stabilization and supportive intensive care if necessary, broad spectrum antibiotic therapy, and extensive surgical debridement of the necrotic tissue [9]. Modern reconstructive techniques, such as skin grafting and flaps, provide reliable coverage of significant tissue defects and acceptable cosmetic results [10]. Despite the advancements in health care over the last decades, the mortality rates of FG still range between 3 and 67% even in developed countries [3]. Several risk scores, like Fournier’s Gangrene Severity Index (FGSI), the Laboratory Risk Indicator for Necrotizing Fasciitis (LRINEC), and the neutrophil–lymphocyte ratio (NLR), have been developed to predict survival and prognosis in FG [11]. To the best of our knowledge, we are the first to report our experiences in treatment of FG in a Swiss tertiary care center and to assess the capability of three different scoring systems (FGSI, LRINEC, and NLR) to predict not only mortality, but also morbidity.

## Methods

Twenty patients treated for FG at the Clinic for Urology of the University Hospital Basel during June 2012 and March 2017 were enrolled in this retrospective study, which was approved by the local IRB (EKNZ number 2017-01336). Patients were identified based on ICD10 coding, and the diagnosis was confirmed by surgical and clinical assessment. Demographic and clinical data, such as age, sex, comorbidities, laboratory and microbiological findings, surgical and antibiotic treatment, supportive measures, reconstructive techniques, and outcomes, were extracted by chart review. Mortality was defined as disease-related death during hospital stay. FGSI [12] was calculated based on clinical (temperature, heart rate, and respiration rate) and laboratory parameters (serum sodium, serum potassium, serum creatinine, serum bicarbonate, hematocrit, and leukocyte count) upon admission. LRINEC [13] was obtained by combining the values for C-reactive protein, leukocyte count, hemoglobin, serum sodium, serum creatinine, and glucose. NLR [14] was calculated as neutrophil–lymphocyte count ratio at admission. We performed prompt radical surgical debridement in all patients and initiated immediate antibiotic treatment. In case of scrotectomy, testes were temporarily placed in medial thigh pockets. Sequential debridement was performed until healthy granulation tissue had developed. Reconstruction of large tissue defect was achieved with skin grafts or flaps. Empiric antibiotic treatment was adjusted according to antibiotic susceptibility testing of the recovered pathogens. Septic patients requiring intensive fluid resuscitation, vasopressors, or mechanical ventilation were treated in intensive care unit (ICU).

All data were analyzed with SPSS 19 (SPSS Inc., Chicago, Illinois, USA). Data on an ordinal or continuous level were analyzed using a non-parametric Mann-Whitney *U* test. Non-normality Spearman’s correlation tests and simple logistic regression tests were performed to determine the association between different independent variables, the severity scores, and mortality. All tests were performed at a significance level of *α* = 0.05.

## Results

**Fig. 2:**
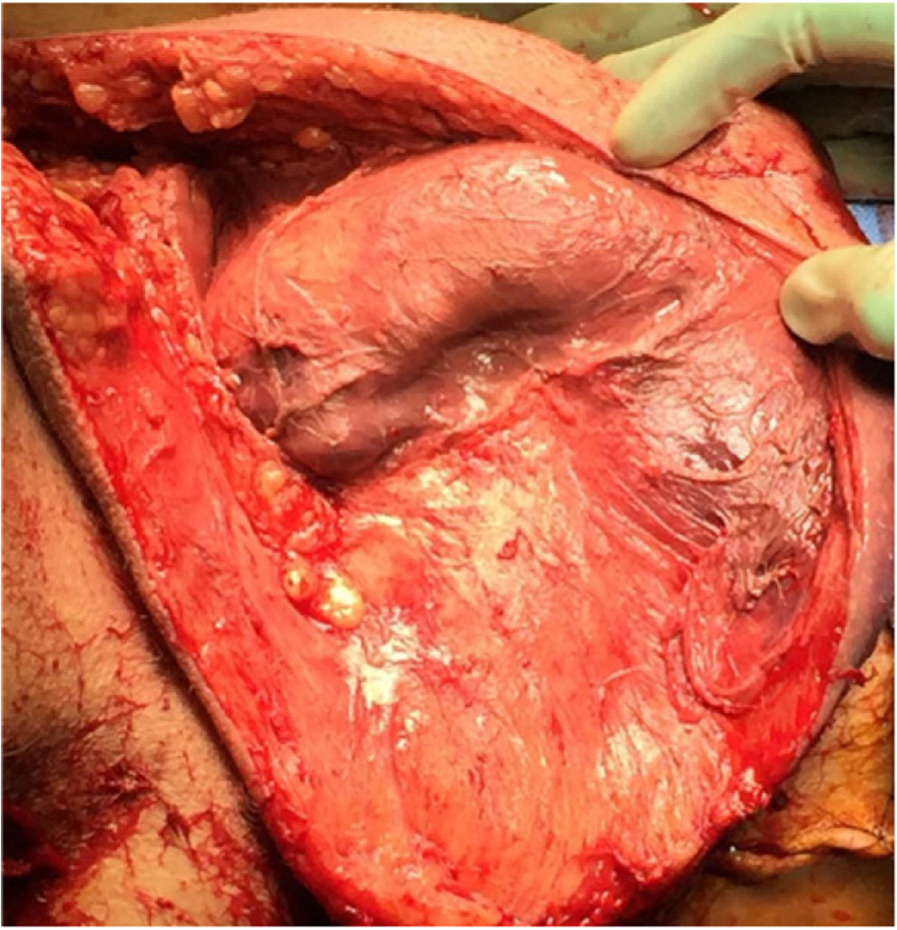
Fournier’s gangrene with complete penile necrosis. Scrotectomy and right orchiectomy already performed

A total of 20 male patients were evaluated. Seventeen patients survived and three patients succumbed to the disease (mortality rate 15%). Median (IQR) age of the patients was 66 (46–73) years. Median (IQR) age of survivors and of non survivors was 64 (43–72) and 84 (67–94) years, respectively. Median (IQR) BMI was 28 (24.8–33.8) kg/m^2^. Predisposing factors like diabetes mellitus, smoking, and renal insufficiency were present in 6/20 (30%), 9/20 (45%), and 9/20 (45%) patients, respectively. Chest X-ray was performed in 8/20 (40%) patients; abdominal CT scan and thoraco-abdominal CT scan were performed in 6/20 (30%) and 3/20 (15%) patients, respectively. For 5/20 (25%) patients, no imaging was performed and diagnosis was based solely on clinical findings. Fifteen (75%) patients required treatment on an ICU with a median (IQR) duration of ICU stay of 3 (2–5) days. None of these parameters correlated significantly with mortality. Nine patients (45%) required mechanical ventilation, with a median (IQR) duration of 2 (1.5–3.5) days. Unilateral orchiectomy was performed in five (35.7%) patients, and one (5%) patient required bilateral orchiectomy. Penectomy was necessary in two (10%) patients due to complete necrosis (Fig. 2). Auxiliary procedures like cystostomy and colostomy were performed in three (15%) and five (25%) patients, respectively. The bacteria cultured from the wound mainly comprised a broad spectrum of aerobic and anaerobic pathogens. *Escherichia coli* was the most common pathogen, identified in 10 (50%) patients. Polymicrobial infections were causative in 18 (90%) patients. Blood cultures were positive in two (12.5%) out of 16 patients with detection of *Klebsiella species* and *Pseudomonas aeruginosa*, respectively. In 17 (85%) patients, the combination of carbapenem antibiotics and clindamycin was used as empiric antibiotic therapy and adjusted according to the cultured bacteria and antibiotic susceptibility testing. Median (IQR) duration of antibiotic treatment was 18 (15–23) days. Extensive and sequential debridements regularly resulted in significant tissue defects. Therefore, nine (45%) patients had to be treated with vacuum-assisted closure (VAC) and five (25%) patients eventually required reconstructive surgery with flaps or skin grafts. The median (IQR) number of required operations was 4 (2–6), and the median (IQR) time to restore the integrity of the body surface was 18 (14–39) days. The mean scores for FGSI, LRINEC, and NLR were 13.0, 9.3, and 45.3 for non-survivors and 7.7, 6.5, and 26.0 for survivors, respectively. Detailed results of the scoring systems are displayed in Table 1. None of the risk scores correlated significantly with mortality, the need of ICU treatment, mechanical ventilation, or the time to restore the integrity of the body surface (*p* > 0.05). However, FGSI (*p* = 0.01), LRINEC (*p* = 0.04), and NLR (*p* = 0.01) correlated significantly with the necessity to perform orchiectomy. We also found a significant correlation for LRINEC (*p* = 0.02) and a borderline significant correlation for NLR (*p* = 0.06) in terms of the need to perform cystostomy but no correlation with the need to perform colostomy. Furthermore, FGSI (*p* = 0.02), LRINEC (*p* = 0.04), and NLR (*p* = 0.03) significantly correlated with the necessity to perform penectomy.

**Table 1:** Risk scores, mortality, and surgical measures in Fournier’s gangrene

Patient	Age	FGSI	LRINEC	NLR	Mortality	Orchiectomy	Penectomy	Reconstructive surgery
1	41	2	2	2.6	No	No	No	Yes
2	37	6	1	6.2	No	No	No	No
3	45	3	2	8.5	No	No	No	No
4	47	6	7	9.3	No	No	No	No
5	66	14	7	16.9	Yes	Yes	No	No
6	87	15	11	107.0	No	Yes	Yes	No
7	63	8	8	27.6	No	No	No	Yes
8	40	7	6	19.0	No	Yes^a^	No	No
9	52	11	11	24.1	No	Yes	No	Yes
10	75	10	8	2.3	No	No	No	No
11	66	10	11	85.2	No	Yes	No	No
12	89	11	9	n.c.	Yes	Yes	No	No
13	72	3	9	21.1	No	No	No	No
14	67	9	4	n.c	No	No	No	No
15	31	13	2	3.7	No	No	No	No
16	65	6	7	21.6	No	No	No	Yes
17	94	14	12	73.7	Yes	No	Yes	No
18	51	6	5	6.7	No	No	No	No
19	72	7	7	38.4	No	No	No	Yes
20	70	8	8	33.6	No	No	No	No

*n.c.* not calculable

^a^Bilateral

## Discussion

**Fig. 3:**
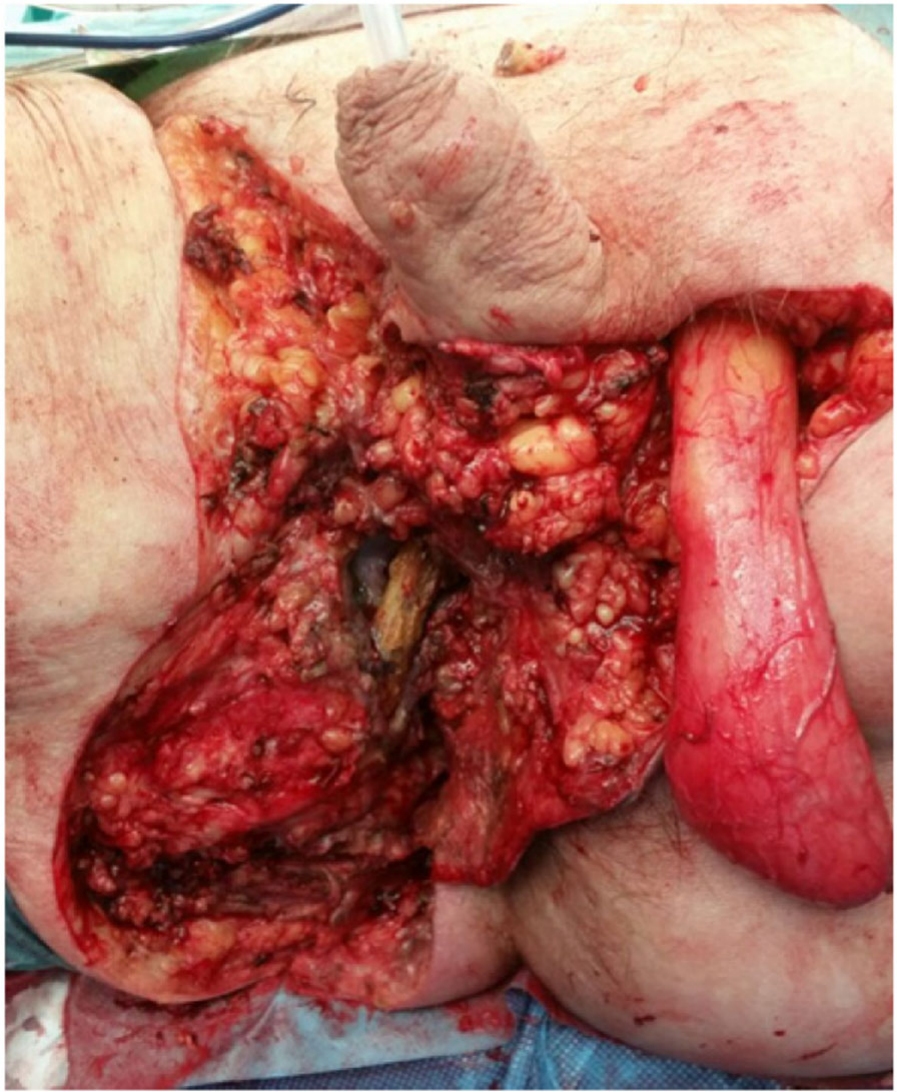
Significant tissue defect after resection of necrotic tissue and right orchiectomy. Exposed pubic bone

Mortality rate in our series was 15%. Two recent reviews [3, 15] reported mortality rates of 3–67%, with higher rates in underdeveloped countries. The high mortality rate that occurs despite major advances in healthcare in general and the implementation of the most modern treatment options and resources at a Swiss tertiary care center reflects the severity of the disease. Yet, with a median age of 66 years (range 46–73), our patients were rather old, as compared to a recent review that reported a median age of 51.8 years (range 47–63 years) [16]. In our series, we could only demonstrate a borderline significance for the correlation of age and mortality, even though it is well known that increasing patient age is a strong independent predictor of mortality [15]. Interestingly, age is not part of the three scoring systems. As age as a variable is easy to assess, we would suggest to include age into scoring systems. Moreover, morbidity of FG is high [3]. Multiple debridements resulted in significant tissue defects (Fig. 3). Therefore, VAC was required in 45% of patients and five patients eventually required reconstructive surgery. Generally, polymicrobial infections comprising a broad spectrum of aerobic and anaerobic pathogen are causative for FG [3], as it was the case in our series. Therefore, the combination of carbapenems and clindamycin is used as standard empiric antibiotic regimen at our institution, adapted from the guidelines of the Infectious Diseases Society of America (IDSA), concerning skin and soft tissue infections [17].

Prediction of the course of the disease is always challenging for the physician. Prognostic indicators like FGSI [12], LRINEC [13], and NLR [14] have been used to determine severity and prognosis of FG (1). The LRINEC had initially been developed to differentiate necrotizing from other soft tissue infections, and the NLR was initially used as sepsis marker, yet both scores were shown to correlate with mortality as well [11]. In the original report, LRINEC scores > 6 were shown to be associated with the likelihood to have necrotizing soft tissue infection [13]. Several reports demonstrated that patients with systemic infection and NLR scores > 10 had a more severe course of the disease [14, 18]. When applying the prognostic index FGSI [12], scores of ≥ 9 suggested a probability of 75% to succumb to the disease, while scores of < 9 were associated with 78% survival. We applied these risk scores on our temporary series, but we could not demonstrate a significant correlation with mortality for any of these prognostic indices, which is most likely due to the small sample size of our study. All non-survivors in our series had FGSI scores > 10. However, several studies have shown controversial results for the accuracy of this test [19–22]. We further assessed whether or not the prognostic indicators correlated with severity of the disease. Patients with a FGSI score ≥ 4 were more likely to require ICU treatment and eventually die than patients with FGSI scores < 4 [11]. We could not reproduce these findings in our cohort. However, we could demonstrate a significant correlation of all risk scores with morbidity in terms of local extent of the disease. Due to spread of necrotizing infection to testicles and penis, the radical and mutilating surgical procedures of orchiectomy (*n* = 6) and penectomy (*n* = 2) were performed to confine further spread of infection. FGSI, LRINEC, and NLR proofed to be predictors for the extent of surgery required, as all of these risk score significantly correlated with orchiectomy, as well as with penectomy. Auxiliary measures like cystostomy or colostomy are regularly required in extensive disease. We found no correlation of the three risk scores with the need to perform colostomy in our cohort, but LRINEC significantly correlated (*p* = 0.02) with the need to perform cystostomy, while NLR showed borderline significance (*p* = 0.06).

To the best of our knowledge, our series is the first to report treatment outcomes of FG in Switzerland. Fournier’s gangrene is a rare disease, which is displayed by our small sample size. In addition to the retrospective nature of the study, the small sample size represents the main limitation of this study. This might explain why we were not able to demonstrate the predictive ability of FGSI, LRINEC, and NLR for mortality. Nevertheless, our report is the first to demonstrate the predictive ability of the risk scores for the local extent of the disease and the required surgical methods in terms of orchiectomy and penectomy. Thus, the application of these risk scores can be a useful adjunct to clinical examination to aid diagnosis and can be helpful in predicting the course of disease. Furthermore, our results highlight that FG remains a potentially fatal disease despite most modern treatment at a Swiss tertiary care center.

## Conclusions

In our series, Fournier’s gangrene was associated with a mortality of 15% despite maximum multidisciplinary therapy at a specialized center. None of the risk score correlated with mortality, but all risk scores correlated with the morbidity of the disease in terms of local extent and the required surgical measures.
